# The relationship between CD204 ^M2^-polarized tumour-associated macrophages (TAMs), tumour-infiltrating lymphocytes (TILs), and microglial activation in glioblastoma microenvironment: a novel immune checkpoint receptor target

**DOI:** 10.1007/s12672-021-00423-8

**Published:** 2021-08-25

**Authors:** Maher Kurdi, Badrah Alghamdi, Nadeem Shafique Butt, Saleh Baeesa

**Affiliations:** 1 Department of Pathology, Faculty of Medicine in Rabigh, King Abdulaziz University, Jeddah, Saudi Arabia; 2 Neuromuscular Unit, Centre of Excellence of Genomic Research, King Abdulaziz University, Jeddah, Saudi Arabia; 3 Department of Physiology, Faculty of Medicine, King Abdulaziz University, Jeddah, Kingdom of Saudi Arabia; 4 Department of Family and Community Medicine, Faculty of Medicine in Rabigh, King Abdulaziz University, Jeddah, Saudi Arabia; 5 Division of Neurosurgery, Faculty of Medicine, King Abdulaziz University, Jeddah, Saudi Arabia

**Keywords:** Glioblastoma, Tumour associated macrophages, CD204, Tumour infiltrating lymphocytes, Microglial activation, Immune check point receptors

## Abstract

**Background:**

Tumour associated macrophages (TAMs) and tumour infiltrating lymphocytes (TILs) are considered dominant cells in glioblastoma microenvironment.

**Aim:**

The purpose of this study was to assess the expression of CD204^+ M2^-polarized TAMs in glioblastomas and their relationship with CD4^+^TILs, Iba^+^microglia, and IDH1 mutation. We also exploreed the prognostic value of these markers on the recurrence-free interval (RFI).

**Methods:**

The expressions of CD204^**+**^TAMs, CD4^**+**^TILs, and Iba1^+^microglia were quantitively assessed in 45 glioblastomas using immunohistochemistry. Kaplan–Meier analysis and Cox hazards were used to examine the relationship between these factors.

**Results:**

CD204^**+**^TAMs were highly expressed in 32 tumours (71%) and the remaining 13 tumours (29%) had reduced expression. CD4^**+**^TILs were highly expressed in 10 cases (22%) and 35 cases (77.8%) had low expression. There was an inverse correlation between CD204^**+**^TAMs and CD4^**+**^TILs, in which 85% of tumours had a high expression of CD204^**+**^TAMs and a low expression of CD4^+^TILs. Nevertheless, there was no significant difference in IDH1 mutation status between the two groups (p = 0.779). There was a significant difference in Iba1^+^microglial activation between IDH1^mutant^ and IDH1^wildtype^ groups (p = 0.031). For cases with a high expression of CD204^+^TAMs and a low expression of CD4^+^TILs, there was a significant difference in RFI after treatment with chemoradiotherapy or radiotherapy (p = 0.030).

**Conclusion:**

Glioblastoma with a dense CD204^**+**^TAMs and few CD4^**+**^TILs is associated with IDH1^wildtype^. These findings suggest that TAMs masks tumour cell and suppress T-cell tumoricidal functions via immunomodulatory mechanisms. Blockade of the CD204-TAM receptor may prevent this mechanism and allow the evolution of TILs.

## Introduction

Glioblastoma is the most common primary malignant tumour of the central nervous system (CNS), accounting for ~ 50% of all primary brain tumours [20]. Despite the standard treatment of surgical resection followed by radiotherapy and adjuvant chemotherapies, it remains the deadliest among all body cancers, having a median survival time of < 15 months [1]. Considering the poor outcome after treatment, there is an urgent need to explore new therapeutic strategies and improve the survival rate. Immune checkpoint receptor inhibitors are a new treatment modality, which is dependent on the array of non-neoplastic cells in the tumour environment.

The glioblastoma microenvironment exhibits a high level of intratumoural heterogeneity, which contains different types of non-neoplastic cells, including immune cells and stromal cells [24]. Tumour associated macrophages (TAMs) and tumour infiltrating lymphocytes (TILs) are considered the dominant infiltrating immune cells in this microenvironment, representing the major content of the tumour mass [2, 10].

TAMs are proangiogenic immune cells that interact with glial cells to promote tumour growth and progression [4]. They are subclassified into two categories: (a) ^M1^-polarized TAMs are induced by interferon gamma to produce proinflammatory molecules including tumour necrosis factor-alpha and are associated with acute inflammatory insults; and (b) ^M2^-polarized TAMs are induced by interleukin-4 stimulation to produce pro-angiogenic growth factors involved in chronic inflammation, tumour growth, and immunosuppression [5, 8, 22] leading to tumour invasion, and the formation of metastatic strings [29]. Clinical data has indicated that a high frequency of ^M2^-polarized TAMs expressing CD163, CD204, and CD206, correlated with a poor prognosis of multiple cancers including melanoma and cancers of the breast, bladder, ovaries, and lungs [5]. In these cancer microenvironments, TILs circulate parallel to TAMs and promote innate immunity. While they have shown a favourable impact on the survival of patients with breast, colorectal, and ovarian cancers [6, 21, 27], their role in the process of gliomagenesis is unclear. TILs expressing CD4^+^ or CD8^+^ represent 0.25% of all cells in the glioblastoma microenvironment [13]. However, CD4^+^T-cells showed an essential role in initiating anti-cancer immune responses, which significantly affects the function of CD8^+^T-cells [9, 15]. Although CD8^+^T-cells are essential for killing tumour cells, their low numbers in the tumour microenvironment are insufficient to have a targeted effect on tumour proliferation and prognosis [16].

CD204, the ^M2^-polarized TAM receptor, is also known as macrophage scavenger receptor 1 (MSR1) [26], preferentially expressed by dendritic cells and macrophages. Few studies have investigated CD204 as an accurate prognostic factor in oesophageal, lung, and breast carcinomas. [17, 23] However, its upregulation was associated with a short survival rate [30]. Recently, it was shown that CD204 associated TAMs among all TAMs are the only independent prognostic factor for gliomas [30]. Although the relationship between TAMs and TILs has not been extensively studied, TILs might synergise with CD204^+^ TAMs as immune checkpoint regulators to inhibit T-cells [30]. When tumour cells evade the immune system via immunomodulatory mechanisms to avoid T-cell inhibitory effects, TAMs (CD204) suppress T-cell tumoricidal functions [3, 18] and promote tumour cell growth and progression. This mechanism may lead to uncontrolled tumour proliferation. Indeed, blockade of the CD204 receptor may prevent this mechanism from occurring, allowing T-cell evolution. In general, these immune checkpoint inhibitors function as T-cell inhibitors and TAM receptor blockers, thereby limiting the threshold immune response. Recently, PD-1^+^ TILs and PD-L1 were reported to be significantly expressed in mouse glioblastomas [7]. However, preclinical trials with anti-PD1/PDL1 blockers showed a lack of efficacy [14].

In this study, we assess the expression of ^M2^-polarized TAMs (CD204) in the glioblastoma microenvironment and their relationship with circulating TILs, mainly CD4^+^TILs, as well as resident microglia. We also investigate the relationship between TAMs and TILs with isocitrate dehydrogenase-1 (IDH1) mutation. The prognostic value of these markers with previous treatment modalities was also explored and correlated with RFI.

## Materials and methods

### Patient stratification

This study included 45 patients, aged between 30 and 70 years, and histologically diagnosed as classical glioblastoma (World Health Organization (WHO)-grade IV Glioma) in the period 2014–2019. This study was approved by the National Biomedical Ethics Committee at King Abdulaziz University (HA-02-J-008) under a general ethical report. All patients underwent complete surgical resection of the tumour followed by postoperative chemoradiation according to the Stupp protocol [28]. Patients’ clinical data were retrieved from hospital records and included patient age at diagnosis, gender, tumour location, types of adjuvant therapy, and recurrence interval. The histological diagnosis was made based on classification of the WHO 2016. The chemoradiotherapy protocol (Stupp protocol) included a total dose of 60 Gy and temozolomide (TMZ), 150–200 mg/m^2^ for 6–12 cycles, was given to all patients at the time of management. Other adjuvant therapies were added to TMZ in some cases, which included (bevacizumab, irinotecan, lomustine, and etoposide). All patients enrolled in this study have passed away.

### Tumour samples

Archival routine formalin-fixed and paraffin-embedded (FFPE) tumour tissues were collected from 45 patients diagnosed histologically with classical glioblastoma. Haematoxylin and Eosin (H&E)-stained sections were re-examined by a certified neuropathologist (MK) to confirm that the histopathological diagnosis was based on the 2016 WHO classification. Four unstained positive-charged slides from each of 45 FFPE tissue blocks were prepared for CD204, CD4, Iba1, and *IDH1*^*R132H*^ immunostaining.

### Immunohistochemistry protocol

First, 4-μm FFPE tissue sections were used for immunohistochemistry (IHC). The IHC assay was performed for different clonal types, directed against human antibodies. The procedure was performed with the ultraView DAB detection Kit from Ventana on a BenchMark XT automated stainer (Ventana, Tucson, AZ, USA). A protocol was established so that the entire assay procedure consisted of deparaffinization with EZ Prep at 75 °C, heat pre-treatment in Cell Conditioning medium (Ag unmasking) (CC1; Ventana) for 60 min and then primary incubation for 16 min at 37 °C. The antibodies were optimized using different dilutions, ranging between 1:100 and 1:300 except for Iba1 was (1:2000). The slides were counterstained with haematoxylin II for 16 min and blueing reagent was used for another 16 min. Then, the slides were removed from the slide stainer and immersed into successive alcohol buffers at different concentrations for 3 min. The antibodies used in this protocol are listed in Table 1.

**Table 1:** List of immunohistochemistry antibodies used in this study

Antibody	Subtype	Description	Staining targets
Anti-CD204	Rabbit polyclonal	Abcam, Cat#217843	TAMs
Anti-CD4	Mouse monoclonal	Ventana, SP34	T-lymphocytes
Anti-Iba1	Rabbit monoclonal	Abcam, EPR16588 Cat# 178846	Resident microglia
Anti-IDH1^R132H^	Mouse monoclonal	Dianova, Clone H09	Tumour cells > 10%

*CD204* macrophage scavenger receptor 1, *TAM* tumour associated macrophages, *Iba1* ionized calcium binding adaptor molecule 1

## Immunohistochemistry assessment

### Quantitative assessment of CD204, CD4, and Iba1 expressions in glioblastoma

**Fig. 1:**
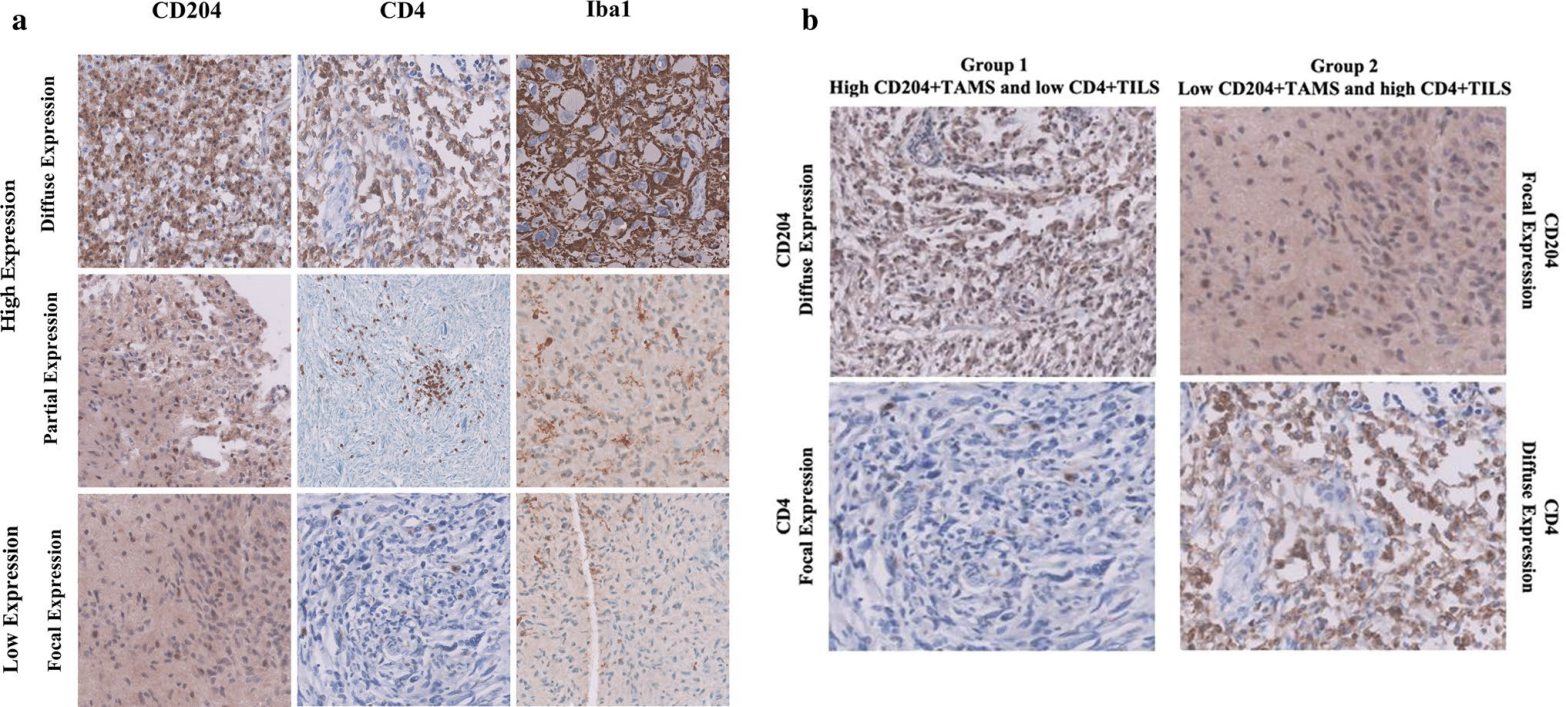
Quantitative assessment of CD204^+^TAMs, CD4^+^TILs, and Iba1^+^ microglial activation in glioblastoma microenvironment using IHC. **a** Staining expression was quantitively analysed and subclassified into categories as described in Table 2, **b** Staining expression of the two groups: group 1 high expression of CD204^+^TAMs with low expression CD4^+^TILs, group 2: low expression of CD204^+^TAMs with high expression CD4^+^TILs

TAMs, TILs, and resident microglia were stained for anti-CD204, anti-CD4, and anti-Iba1 expressions, respectively. Each group of cells was evaluated separately. A single focal area per patient sample, rich in tumour cells and containing sufficient aggregate TAMs, TILs, or resident microglia, was quantitively analysed under light microscopy using high-power (40 ×) magnification (Fig. 1a, b). This area was selected by an expert neuropathologist (MK). The positively stained cells and total cells, including positively and negatively stained cells, were counted manually using labelling indices. For example, CD204^+^TAMs and the total cells (stained TAMs and unstained TAMs) were counted manually, and the labelling index was assessed using the following equation:

$${\text{Labelling index}} (\%) = [(CD204+stained TAMs)/(total cells) \times100].$$

$${\text{Labelling index}} (\%) = [(CD4+stained TILs)/(total cells) \times 100].$$

$${\text{Labelling index}} (\%) = [(Iba1+stained microglia)/(total cells) \times 100].$$

The staining pattern was categorized as (i) diffusely expressed, (ii) partially expressed, (iii) focally expressed, and (iv) not expressed. Diffuse and partial expressions were considered to indicate “high or increased expression” whereas focal expression and not expressed were considered to indicate “low or reduced expression” (Fig. 1a, b). The labelling index (%) was assessed using a scoring system shown in Table 2.

**Table 2:** Quantitative expression of CD204^+^TAMs, CD4^+^TILs, and Iba1^+^Microglia immunostaining using labelling indices

Expression	Labelling index (%)^a^
No expression	0
Focal expression	> 0–19
Partial expression	> 19–49
Diffuse expression	≥ 50

Labelling index (%) = [(^+^*stained cells)/(total cells)* × *100*]. Diffuse and partial expression was considered “high expression” whereas focal and no expression were considered “low expression”

^a^For statistical analysis, the scores were divided by 100

### Assessment of IDH1^*R132H*^ expression in glioblastomas

We assessed *IDH1*^*R132H*^ glial tumour cells that only harboured a point mutation. Sections with > 10% of tumour cells positively stained were defined as IDH1^mutant^.

## Statistical methods

Data were described as frequencies and percentages. Pearson’s Chi-Square test was used to explore the relationship between CD204^+^TAMs, CD4^+^TILs, Iba1-associated macrophages, and IDH1^R132H^ mutation. Hazard regression plot (univariable and multivariable) was used to assess the relationship between CD204 and CD4 expression and treatment modalities on RFI. Kaplan–Meier curves and log-rank test were used to compare the distribution of RFI among glioblastoma cases with different quantities of CD204^+^TAMs and CD4^+^TILs. The RFI was defined as the period from the beginning of adjuvant therapy after surgical resection to the possible first date of recurrence. All statistical analyses in this study were performed using IBM SPSS1 ver. 24 (SPSS Inc., Chicago, IL, USA) and “R” statistical software programs.

## Results

**Fig. 2:**
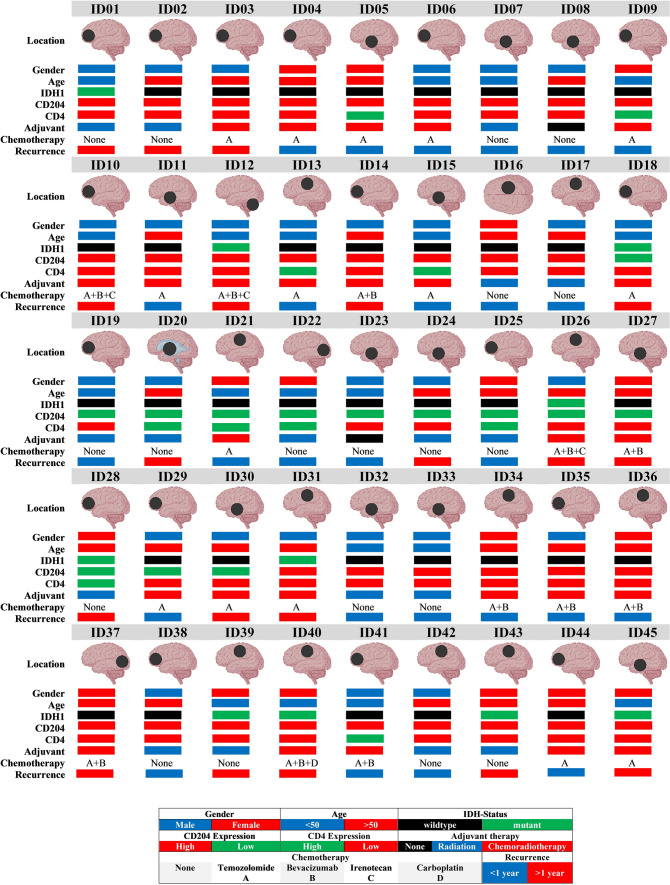
Clinical and biological information of 45 glioblastoma patients enrolled in the study including age, gender, IDH1 mutation, expression of CD204^+^TAMs and CD4^+^TILs, and their treatment protocol including chemotherapeutic agents, and RFI

This study included 45 patients diagnosed with classical glioblastoma (mean age: 51 years (± 21 years); 28 males (62.2%) and 17 females (37.8%)). Approximately 40% (n = 18) of the tumours were in the frontal lobe followed by the temporal lobe (26.7%, n = 12) (Table 3). The rest of the cases (33.3%) were in the parietal, occipital, intraventricular, and posterior fossa regions. IDH1 mutation was found in 10 cases (22.2%) and wildtype IDH1 was detected in the remaining 35 cases (77.8%). The expression of CD204-associated TAMs, CD4-associated TILs, and Iba1-associated microglia was examined in 45 glioblastoma patients who received different treatment modalities (Fig. 2). Approximately 57.8% (n = 26) of patients received standard chemoradiotherapy, 17 patients (37.8%) received only radiation, and two patients refused to receive any treatment modalities because of their poor health status. Of those who received chemotherapy, 15 (57.7%) received only TMZ and the remaining 11 patients (42%) received TMZ with additional chemotherapeutic agents (bevacizumab, irinotecan, lomustine, or etoposide). The mean RFI (in) was 14.0 ± 10.2 months.

**Table 3:** Demographic data of the 45 glioblastoma patients enrolled in the study including clinical and biological information

	Overall (N = 45)
Age	
Mean (SD)	51.5 (21.2)
Range	30.0–70.0
Sex	
Female	17 (37.8%)
Male	28 (62.2%)
Tumour location	
Frontal	18 (40%)
Temporal	12 (26.7%)
Parietal	11 (24.4%)
Occipital	2 (4.4%)
Lateral ventricle	1 (2.2%)
Posterior fossa	1 (2.2%)
IDH1 status	
IDH-^mutant^	10 (22.2%)
IDH-^wildtype^	35 (77.8%)
CD4	
High expression	10 (22.2%)
Low expression	35 (77.8%)
CD204	
High expression	32 (71.1%)
Low expression	13 (28.9%)
Iba1	
High expression	40 (88.9%)
Low expression	5 (11.1%)
Adjuvant	
Chemoradiotherapy	26 (57.8%)
Radiation	17 (37.8%)
None	2 (4.4%)
Chemotherapy	
Temozolomide	15 (57.7%)
Temozolomide with adjuvants	11 (42.3%)
Recurrence interval (months)	
Mean (SD)	14.1 (10.2)
Range	0.0–37.4

### Relationship between CD204^+^TAMs and CD4^+^TILs in glioblastoma microenvironment

**Fig. 3:**
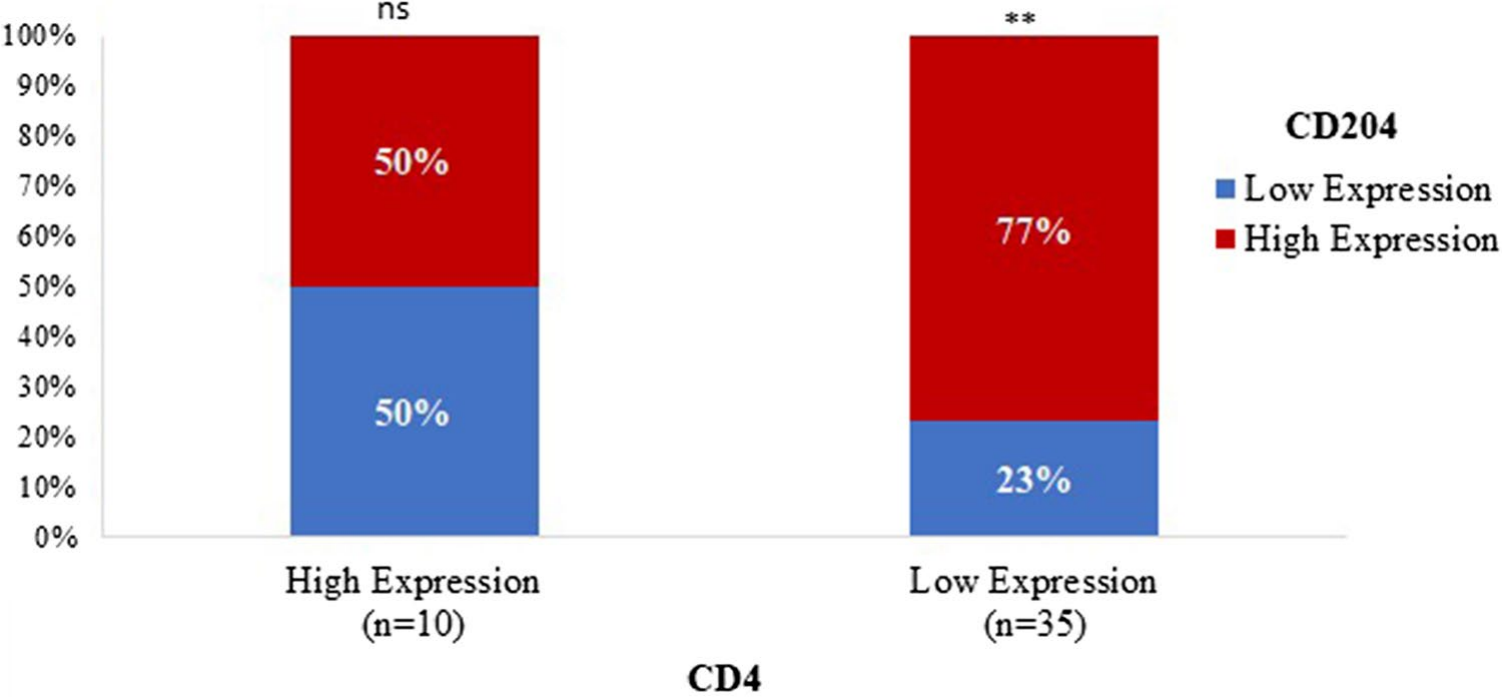
A diagram illustrating the inverse correlation between CD204^+^TAMs and CD4^+^TILs. Approximately 77% of 35 tumours with a low expression of CD4^+^TILs had a high expression of CD204^+^TAMs, and 23% of cases had a low expression of CD204^+^TAMs

**Fig. 4:**
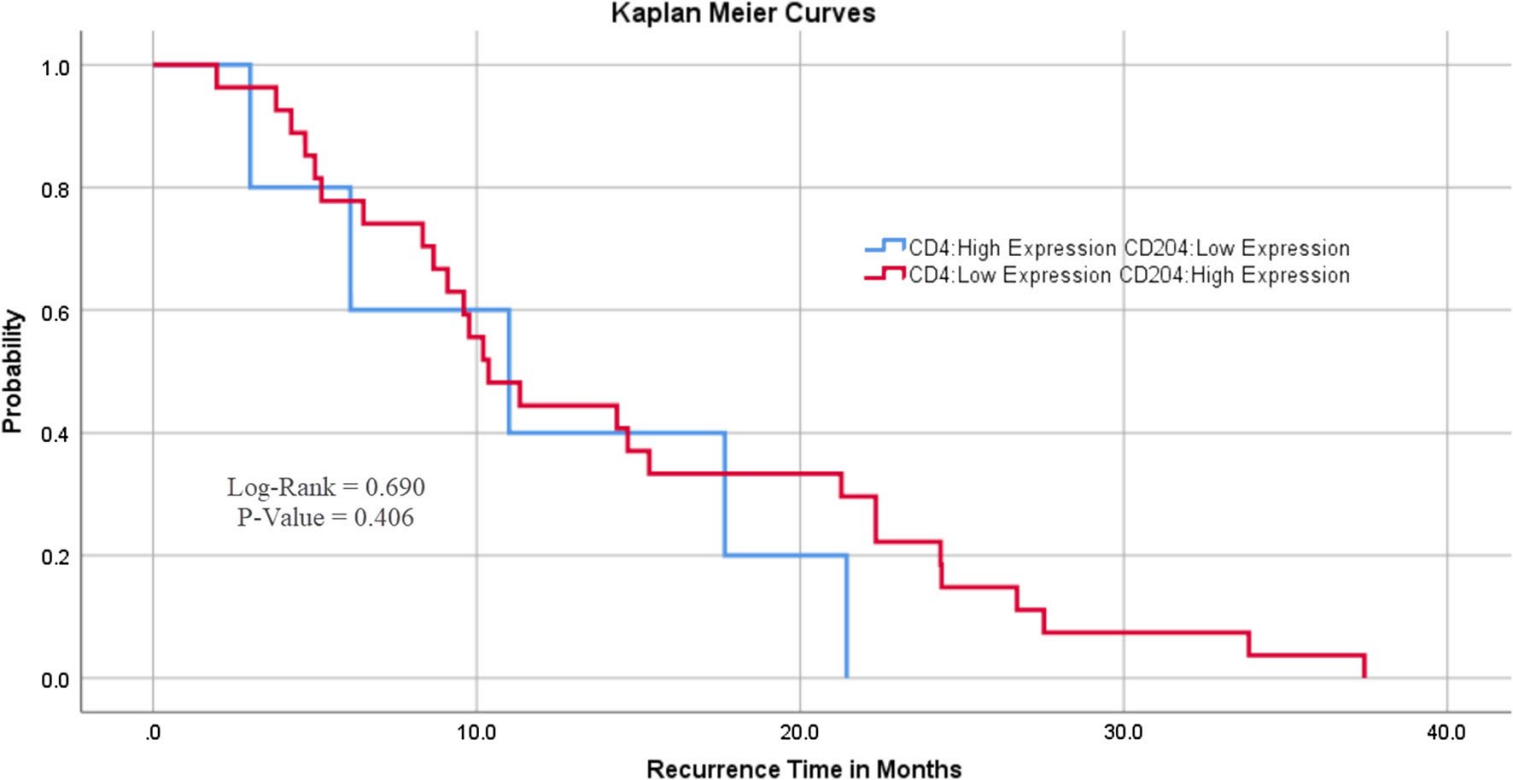
The relationship between CD204^+^TAMs and CD4^+^TILs with the RFI. Patients with a high expression of CD204^+^TAMs and a low expression of CD4^+^TILs had late recurrence

CD204^+^TAMs were highly expressed in 32 glioblastomas (71%) and the remaining 13 tumours (29%) showed reduced expression. CD4^+^TILs were highly expressed in 10 cases (22%) of glioblastomas, and 35 cases (77.8%) showed low expression. There was an inverse correlation between CD204^+^TAMs and CD4^+^TILs. Approximately 85% of the tumours had a high expression of CD204^+^TAMs with a low expression of CD4^+^TILs. Of 13 cases with a low expression of CD204^+^TAMs, only five cases (38.5%) were associated with a high expression of CD4^+^TILs. Although this finding was scientifically consistent, it was statistically insignificant (p = 0.095) (Table 4). Moreover, approximately 77% of 35 tumours with a low expression of CD4^+^TILs had a high expression of CD204^+^TAMs whereas 23% of tumours had a low CD204^+^TAMs expression (Fig. 3). Glioblastomas with a high expression of CD204^+^TAMs and a low expression of CD4^+^TILs were associated with late recurrence. The mean recurrence for cases with high expression of CD204^+^TAMs and a low expression of CD4^+^TILs was 14.5 months while the mean recurrence for cases with low expression of CD204^+^TAMs and a high expression of CD4^+^TILs was 11.8 months. Nevertheless, there was no significant difference in RFI between the two groups (Fig. 4).

**Table 4:** Relationship between CD204^+^TAMs and CD4^+^TILs in glioblastomas

	CD204^+^TAMs			
	High expression (n = 32)	Low expression (n = 13)	Total (n = 45)	p-value
CD4^+^TILs				0.095^1^
High expression	5.0 (15.6%)	5.0 (38.5%)	10.0 (22.2%)	
Low expression	27.0 (84.4%)	8.0 (61.5%)	35.0 (77.8%)	

There was an inverse correlation between CD204^+^TAMs and CD4^**+**^TILs. We found 84.4% of the tumours had a high expression of CD204^+^TAMs with a low expression of CD4^+^TILs. Of 13 cases with a low expression of CD204^+^TAMs, only 5 cases (38.5%) were associated with a high expression of CD4^+^TILs

^1^Pearson’s Chi-squared test

### Relationship between CD204^+^TAMs and CD4^+^TILs with IDH1^mutant^ glioblastomas

**Fig. 5:**
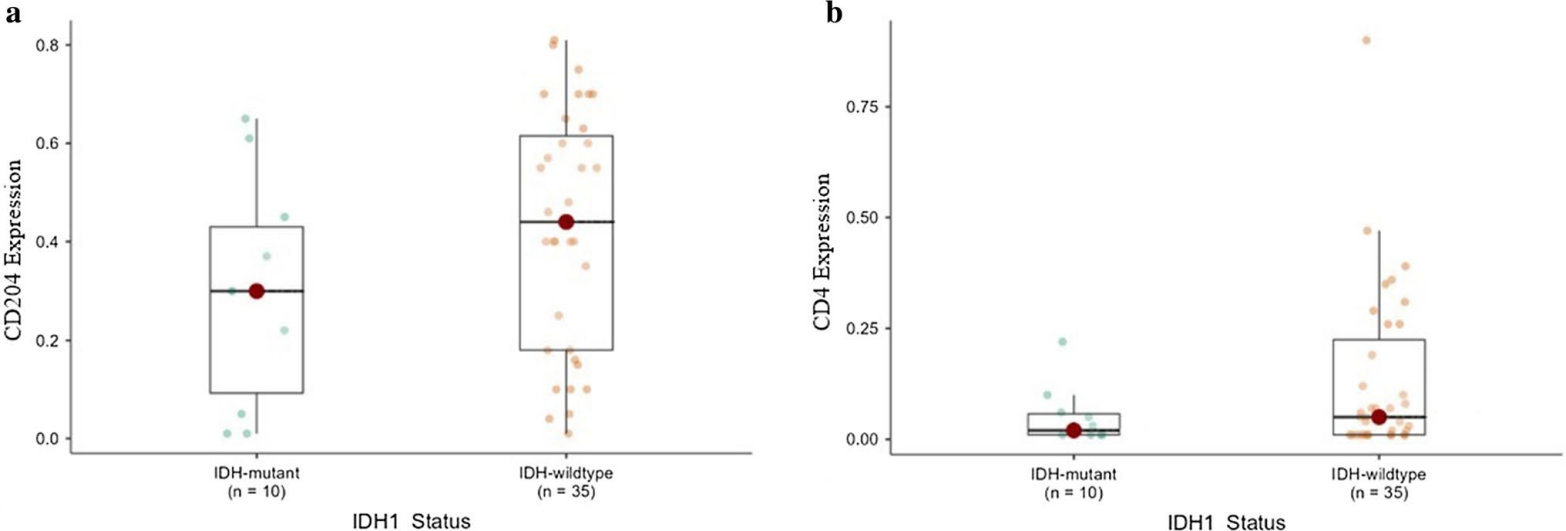
Correlation between CD204^+^TAMs and CD4^+^TILs with IDH1 mutation in glioblastomas. A high expression of CD204^+^TAMs with a low expression of CD4^+^TILs was associated more with IDH1^wildtype^ glioblastomas

Although highly expressed CD204^**+**^TAMs were associated with IDH1^wildtype^ glioblastomas (n = 25) when compared with IDH1^mutant^ glioblastomas (n = 7), there was no significant difference between the two groups (p = 0.930) (Table 5; Fig. 5a). A low expression of CD4^+^TILs was associated more with IDH1^wildtype^ glioblastomas (n = 26) compared with IDH1^mutant^ glioblastomas (n = 9). However, there was no significant difference between the two groups (p = 0.292) (Table 5; Fig. 5b).

**Table 5:** Relationship between CD204^+^TAMs, CD4^+^TILs, and Iba1^+^Microglia with IDH1^mutant^ glioblastomas

	IDH1^mutant^ (n = 10)	IDH1^wildtype^ (n = 35)	Total (n = 45)	p-value
CD4^+^TILs				0.292^1^
High expression	1.0 (10.0%)	9.0 (25.7%)	10.0 (22.2%)	
Low expression	9.0 (90.0%)	26.0 (74.3%)	35.0 (77.8%)	
CD204^+^TAMs				0.930^1^
High expression	7.0 (70.0%)	25.0 (71.4%)	32.0 (71.1%)	
Low expression	3.0 (30.0%)	10.0 (28.6%)	13.0 (28.9%)	
Iba1^+^Microglia				0.031^1^
High expression	7.0 (70.0%)	33.0 (94.3%)	40.0 (88.9%)	
Low expression	3.0 (30.0%)	2.0 (5.7%)	5.0 (11.1%)	

Although highly expressed CD204^+^TAMs were associated with IDH1^wildtype^ glioblastomas compared with IDH1^mutant^ glioblastomas, there was no significant difference between the two groups (p = 0.930). Iba1^+^microglia were significantly expressed in IDH1^wildtype^ glioblastomas (p = 0.031)

^1^Pearson’s Chi-squared test

Despite the high expression of CD204^+^TAMs with low CD4^+^TILs expression in 20 IDH1^wildtype^ glioblastomas compared with 7 IDH1^mutant^ glioblastomas, this did not reach statistical significance (p = 0.779) (Table 6).

**Table 6:** The inverse relationship of CD204^+^TAMs and CD4^+^TILs with IDH1 mutation

	IDH1 status			
	IDH1^mutant^ (n = 8)	IDH1^wildtype^ (n = 24)	Total (n = 32)	P-value
CD4^+^TILs and CD204^+^TAMs				0.779^1^
CD204 high, CD4 low	7.0 (87.5%)	20.0 (83.3%)	27.0 (84.4%)	
CD204 low, CD4 high	1.0 (12.5%)	4.0 (16.7%)	5.0 (15.6%)	

Cases with highly expressed CD204^+^TAMs and low CD4^+^TILs expression were detected more often in IDH1^wildtype^ glioblastomas than IDH1^mutant^ glioblastomas; however, there was no statistical significance between the groups (p = 0.779)

^1^Pearson’s Chi-squared test

### Iba1^+^microglia in the glioblastoma microenvironment

Iba1-associated microglial activation was high in the glioblastoma microenvironment (90%, n = 40). Regarding Iba1-microglial activation, Iba1 expression was significantly increased in IDH1^wildtype^ glioblastomas compared with IDH1^mutant^ glioblastomas (p = 0.031) (Table 5). Regarding CD204, a high expression of CD204^+^TAMs was associated with Iba1^+^microglia, although this did not reach statistical significance (p = 0.104).

### Relationship between CD204^+^TAMs and RFI in glioblastoma patients receiving postsurgical chemoradiotherapy

The univariate hazard ratio (HR) for a low expression of CD204^+^TAMs was 0.75 (1/0.75 = 1.33), indicating a 1.33 times significantly lower chance of tumour recurrence compared with patients with a high expression of CD204^+^TAMs (p = 0.039). The multivariate HR for a low expression of CD204^+^TAMs was 0.44 (1/0.44 = 2.27) indicating a 2.27 times significantly lower chance of tumour recurrence compared with patients with a high expression of CD204^+^TAMs, when controlled for CD204 and subtype of treatment modality (p = 0.038) (Table 7).

**Table 7:** Relationship between CD204^+^TAMs and RFI in glioblastoma patients receiving chemoradiotherapy

Dependent: recurrence	All	HR (univariable)	HR (multivariable)
CD204^+^TAMs			
High expression	32 (100)		
Low expression	13 (100)	0.75 (0.39–1.45, p = 0.039)	0.44 (0.20–0.96, p = 0.038)
Adjuvant			
Chemoradiotherapy	26 (100)		
Radiotherapy	17 (100)	1.94 (0.98–3.8, p = 0.056)	1.85 (0.92–3.73, p = 0.084)

Univariate or multivariate HR indicated a 0.5 times lower chance of tumour recurrence if radiotherapy was used individually compared with combination therapy. These findings were statistically insignificant (p = 0.056, p = 0.084)

Univariate or multivariate HR for use of radiation therapy alone for the treatment of glioblastoma was 1.94 and 1.85, respectively. This indicates a 0.5 times lower chance of tumour recurrence if radiotherapy is used individually compared with combination therapy. However, the HR for univariate and multivariate regressions were statistically insignificant (p = 0.056, p = 0.084, respectively) (Table 7).

**Fig. 6:**
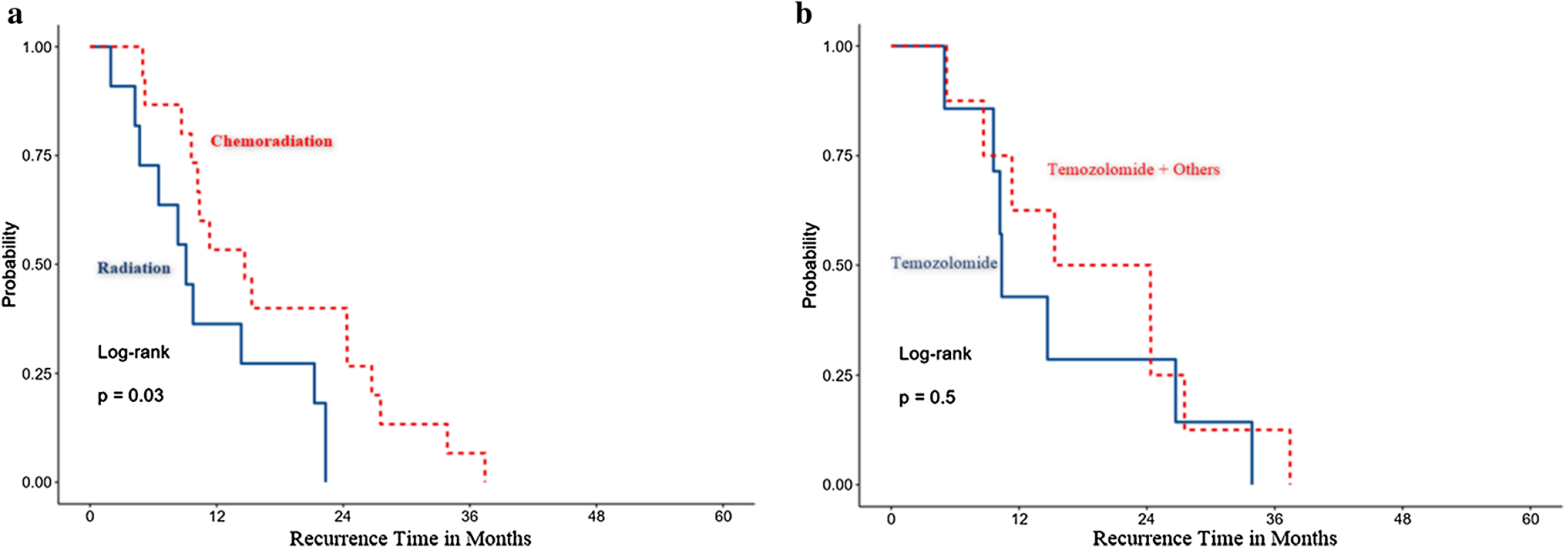
The impact of treatment modalities on the RFI in glioblastoma patients with a high expression of CD204^+^TAMs and a low expression of CD4^+^TILs. **a** There was a significant difference in the RFI of cases with a high expression of CD204^+^TAMs and a low expression of CD4^+^TILs when treated with chemoradiotherapy or radiotherapy. **b** There was no significant difference in the RFI of cases with a high expression of CD204^+^TAMs and a low expression of CD4^+^TILs when treated with TMZ or TMZ with additional adjuvants

There was a significant difference in tumour RFI between chemoradiotherapy and radiotherapy (p = 0.030) in glioblastoma cases with a high expression of CD204^+^TAMs and a low expression of CD4^+^TILs (Fig. 6a).

### Relationship between CD204^+^TAMs and RFI in glioblastoma patients who received specific types of adjuvant chemotherapy

**Fig. 7:**
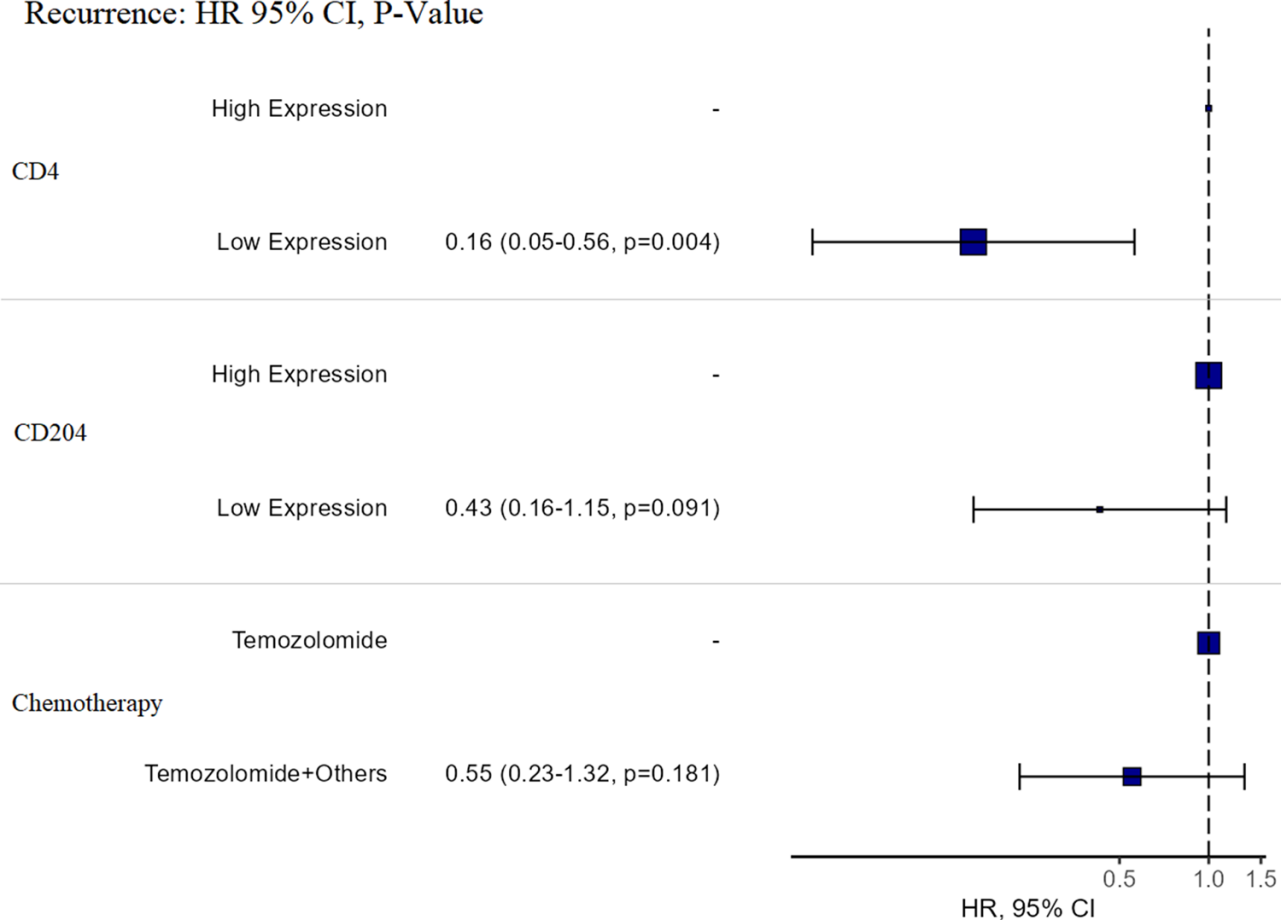
Relationship between CD204^+^TAMs and CD4^+^TILs with the RFI in glioblastoma patients who received specific types of chemotherapy using the multivariate HR. There was a lower chance of tumour recurrence in cases with a low expression of CD204^+^TAMs. There was a lower chance of tumour recurrence after treatment with TMZ and adjuvants

The univariate HR for a low expression of CD204^+^TAMs was 0.56 (1/0.56 = 1.78) indicating a 1.78 times lower chance of tumour recurrence compared with patients with a high expression of CD204 + TAMs. However, this was not statistically significant (p = 0.226). The multivariate HR for a low expression of CD204^+^TAMs was 0.43 (1/0.43 = 2.32) indicating a 2.32 times lower chance of tumour recurrence compared with patients with a high expression of CD204^+^TAMs when controlled for CD4 and chemotherapies; however, this difference did not reach statistical significance (p = 0.091) (Fig. 7).

The univariate HR for the use of TMZ with additional adjuvants was 0.50 (1/0.5 = 2.0) indicating a 2.00 times lower chance of tumour recurrence compared with TMZ only therapy. The multivariate HR for the use of TMZ with additional adjuvants was 0.55 (1/0.55 = 1.81) indicating a 1.81 times lower chance of tumour recurrence compared with TMZ only therapy. The HR for univariate and multivariate regressions did not reach statistical significance (p = 0.102, p = 0.181, respectively) (Fig. 7).

There was no significant difference in RFI between treatment with TMZ and TMZ with additional adjuvants (p = 0.50) in glioblastoma cases with a high expression of CD204^+^TAMs and a low expression of CD4^+^TILs (Fig. 6b).

## Discussion

The glioblastoma microenvironment consists of different cellular heterogeneities including neoplastic cells and non-neoplastic cells that are segmented as tumour niches where treatment-resistant glioma stem cells are localized [12]. TAMs are considered the dominant non-neoplastic immune cells in this microenvironment and are responsible for tumour growth and proliferation. They originate from resident microglia and bone derived cells, produce metalloproteinase 9, regulate immunosuppression, and initiate TAMs-tumour reprogramming [11]. It is still unclear whether the tumour cells reprogram TAMs to a pro-tumorigenic phenotype, or the opposite. However, both are considered targets for adjuvant immunotherapy.

**Fig. 8:**
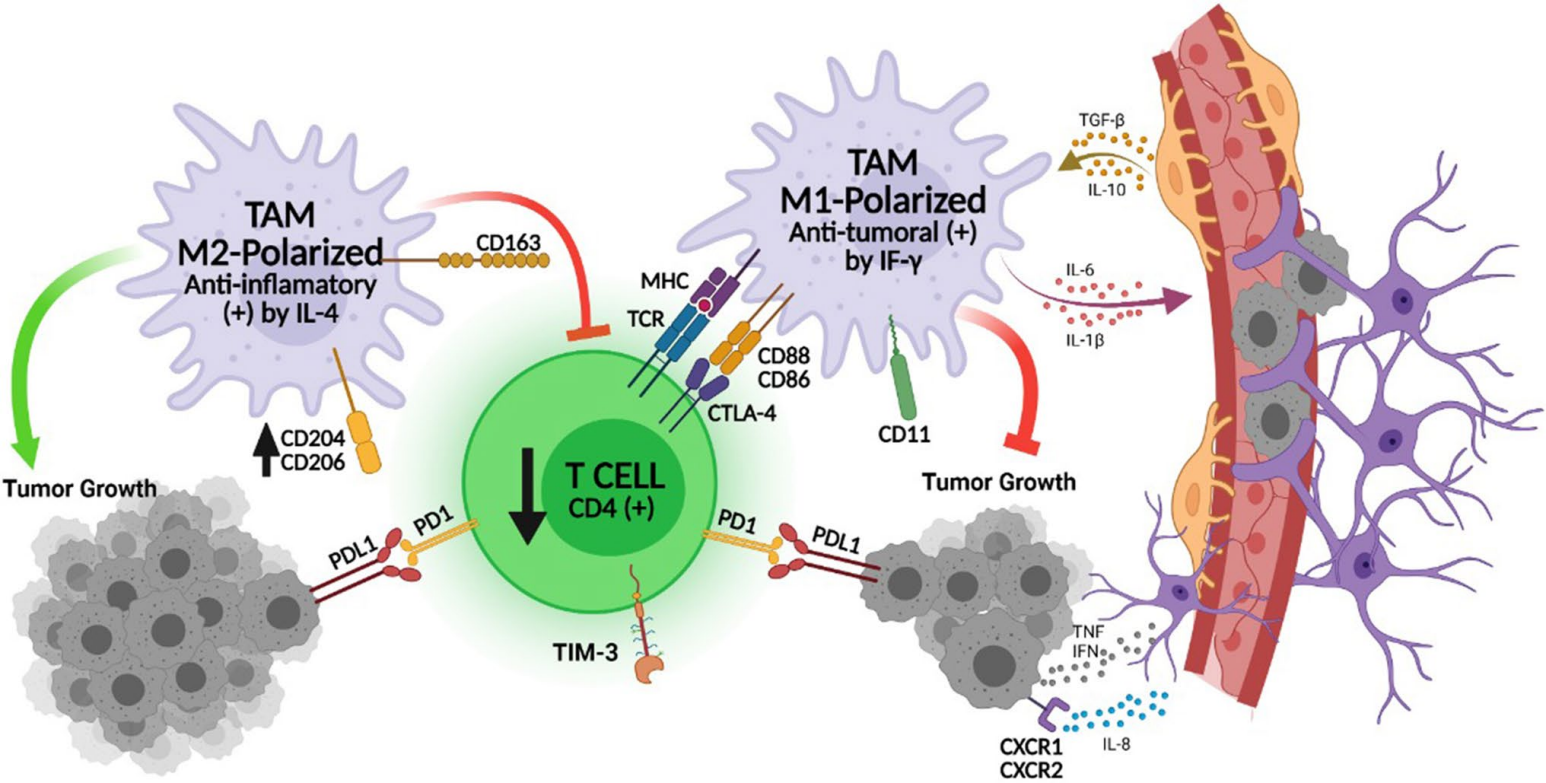
The diagram illustrates the two subclasses of TAMs and the effect of ^M2^-polarized TAMs on tumour proliferation and T-cell function. When tumour cells evade the immune system via the immunomodulatory effect of TAMs avoiding T-cell inhibitory actions, ^M2^-polarized TAMs suppress T-cell tumoricidal functions, which promotes uncontrolled tumour proliferation. This occurs when CD204, a ^M2^-polarized TAMs receptor, is highly expressed in the tumour microenvironment. Theoretically, CD204^+^TAMs modulate tumour cell proliferation and angiogenesis, and inhibit TILs tumoricidal function

Two subclasses of TAMs have been identified: ^M1^-polarized “anti-tumoural” TAMs and ^M2^-polarized “anti-inflammatory” TAMs (Fig. 8). Unlike ^M1^-polarized TAMs, ^M2^-polarized TAMs have an immunomodulatory role, which promotes tumour progression and angiogenesis via the secretion of growth factors leading to the formation of metastatic niches [22, 29]. One of the newly discovered ^M2^-polarized TAMs in the tumour microenvironment are CD204-linked TAMs [26, 30]. CD204 is also known as macrophage scavenger receptor 1 (MSR1) [26], preferentially expressed by dendritic cells and macrophages. Studies revealed its upregulation in several body cancers was associated with a short survival rate [30]. Recently, it was shown that CD204 is the only independent prognostic factor for gliomas among all TAMs [30]. Although its relationship with TILs has not been extensively investigated, they might synergise with CD204^+^TAMs as immune checkpoint regulators that inhibit T-cells [30]. This synchronous correlation has not been explored and further studies should be initiated.

When tumour cells evade the immune system via immunomodulatory effects, ^M2^-polarized TAMs suppress T-cell tumoricidal functions, which promotes uncontrolled tumour proliferation [3] (Fig. 8). Theoretically, TAMs mask tumour cells and suppress T-cell tumoricidal function. Hence, T-cells may not be able to help tumour cells to evade the immune system. This will lead TAMs accumulating in the microenvironment with less T-cells evolution. In our study, we revealed that CD204^+^TAMs were highly expressed in the microenvironment of 71% of our glioblastoma samples whereas there were fewer CD4^+^TILs in the glioblastoma microenvironment (Table 3). This imbalance cannot be considered when CD4^+^TILs are highly expressed in the microenvironment. This observation emphasizes that high level of CD204^+^TAMs associated with late recurrence because of the previously mentioned theory.

All previous studies in this field were performed using various human glioblastomas lines and models, without considering the genetic makeup of the tumours (such as IDH1 mutations) making it difficult to determine whether each identified target was a pan-TAM target or was specific for a specific model. Because IDH1 mutation is considered a major prognosticator in glioblastoma, we report a correlation between CD204^+^TAMs and CD4^+^TILs with IDH1 mutation. Cases with a high expression of CD204^+^TAMs and a low expression of CD4^+^TILs had late tumour recurrence (Fig. 4) and was more frequently present in IDH1^wildtype^ type glioblastomas (77%) (Fig. 5). Furthermore, this suggests that a large number of CD204^+^TAMs in the tumour microenvironment may promote tumour cell proliferation and angiogenesis, although they also suppress T-cell tumoricidal effects (Fig. 8).

As mentioned earlier, there was a synergetic relationship between TAMs and TILs, which affected tumour cell proliferation. CD4^+^TILs had an essential role in initiating anti-cancer immune responses that significantly affected the function of CD8^+^TILs against tumour cells [9]. Although CD8^+^TILs are essential for killing tumour cells, their low numbers in the tumour microenvironment are insufficient to affect tumour growth. The low numbers of CD8^+^TILs might be related to their inability to cross the blood–brain barrier efficiently. Han et al. found that the number of CD8^+^TILs was inversely correlated with tumour grade whereas the number of CD4^+^TILs was positively correlated with tumour grade [13]. This theory is contradictory in glioblastoma. In our study, the tumour microenvironment contained very few CD4^+^TILs (77.8%) because there were high numbers of TAMs that inhibit T-cell recruitment. The presence of low numbers of CD8^+^TILs in the glioblastoma microenvironment is associated with a poor outcome because cytotoxic lymphocytes are unable to kill tumour cells while CD4^+^TILs are completely suppressed. This only occurs when CD204^+^TAMs are infiltrated the tissue, which allows tumour cells to evade the immune system. Therefore, the prognostic value of T-cells in glioblastoma is still unclear; however, their roles may be strongly linked with TAMs.

Based on these findings, we concluded that malignant glial cell proliferation can be prevented if CD204^+^TAM receptors are blocked, which in turn, allows CD4^+^TIL evolution and enhanced CD8^+^TIL toxicity. As a result, CD204^**+**^TAM receptors may be a suitable target for TAMs blockade. In recent clinical trials, some immune checkpoint inhibitors were used as TAMs receptor blockers, which stimulated CD4^+^TILs activity and limited the threshold of the immune response. Cytotoxic T-lymphocyte- associated antigen-4 (CTLA-4), programmed cell death-1 receptor (PD-1), and T-cell inhibitory receptor (TIM-3) are expressed on T-cells and mediate inhibitory effects by interacting with their ligands on tumour cells or TAMs [19] (Fig. 8). It was recently reported that the number of PD-1^+^TILs as well as PD-L1 expression were significantly increased in glioblastoma, suggesting the use of immune checkpoint blockade to interrupt the PD-1/PD-L1 pathway [10]. Although preclinical trials with anti-PD1/PDL1 blockers (nivolumab/pembrolizumab) were not favourable, their use with an anti-CTLA4 blocker (ipilimumab) significantly prolonged the survival rate in glioblastoma [14]. However, the results were not promising in human phase III trials [14, 25]. T cell immunoglobulin and mucin domain-containing protein 3 (TIM-3) were independent prognostic factors in glioblastoma and might be an alternative potential therapy to PD1/PDL1 inhibitors [19]. Currently, their efficacy is still being investigated. Yuan et al. reported a positive correlation between CD204 expression and other immune checkpoints such as PD‐1, suggesting potential synergy between CD204^+^TAMs and immune checkpoint regulators to limit T-cell functions [30].

The association between TAMs and current treatment modalities (chemotherapy and radiotherapy) with tumour recurrence has not been investigated. Here, we explored the impact of chemoradiotherapy on tumour recurrence in glioblastoma with different CD204^+^TAM expressions, using univariate and multivariate HR regression plots (Table 7; Fig. 7). The recurrence of glioblastoma was delayed if CD204^+^TAMs were highly expressed in the tumour microenvironment. However, if CD204^+^TAMs were highly expressed with a low expression of CD4^+^TILs, tumour recurrence was reduced after combination therapies (chemoradiation) (Fig. 6a). This suggests that chemoradiation may have an important role in reducing TAMs in the tumour microenvironment (Fig. 6a). However, the mechanism involved requires further investigation. Furthermore, different types of chemotherapies had no effect on the glioblastoma tumour RFI when there was a high expression of CD204^+^TAMs and a low expression of CD4^+^TILs (Fig. 6b). This suggests that specific types of chemotherapeutic agents have no significant effect on the RFI, indicating the use of TMZ or TMZ with other chemotherapeutic agents would have similar effects on the RFI.

## Conclusions

The aim of our study was to investigate the synergetic mechanisms of CD204^+^TAMs and CD4^+^TILs in glioblastoma microenvironment and to determine how these immune cells affect tumour recurrence. To date, immune checkpoint inhibitor biomarkers are used as surrogates for antitumor T-cell responses, which include T-cell counts in the tumour microenvironment. ^M2^-TAMs (CD204) modulate tumour cell proliferation and inhibit T-cell evolution. All these factors affect the tumour recurrence status. Understanding the mechanism of TAMs in glioma progression might help develop therapies that suppress the tumour-progressing effects of TAMs.

## Data Availability

The data that support the findings of this study are available from the corresponding author (MK) upon request.
